# Measurement of CD4^+^ T cells in point-of-care settings with the Sysmex pocH-100i haematological analyser

**DOI:** 10.1111/j.1751-553X.2007.01017.x

**Published:** 2009-04

**Authors:** C BRIGGS, S MACHIN, M MÜLLER, W HAASE, K HOFMANN, F FORSTREUTER, R HINZMANN

**Affiliations:** * Department of Haematology, University College London HospitalLondon, UK; † Labor FennerHamburg, Germany; ‡ Medilys, Asklepios Klinik NordHamburg, Germany; § Sysmex Europe GmbHNorderstedt, Germany

**Keywords:** HIV, CD4 lymphocyte count, pocH-100i, resource-limited settings, antiretroviral therapy

## Abstract

The decision to provide antiretroviral therapy to HIV-positive patients mainly depends on the CD4^+^ T-cell count, with therapy indicated at a cut-off value of <350–200 CD4^+^ T cells/*μ*l blood. Monitoring patients is still a major problem in countries with limited resources where blood samples often have to be transported over long distances to regional referral centres in which the count can be performed on flow cytometers. We have evaluated a newly developed simple and inexpensive method for CD4^+^ T-cell quantification. It is a variation of the Invitrogen T4 Quant kit, with manual isolation of nuclei from CD4^+^ T cells and subsequent counting on the small haematology analyser pocH-100i, Sysmex. We have demonstrated that this new method is highly reproducible and gives stable and linear results over a wide range of CD4^+^ T-cell concentrations. Method comparison to two different flow cytometers showed excellent correlation with concordances of about 93%. Overall, this method is rapid, easy to perform and offers a good reliable alternative to measurement by flow cytometry. The pocH-100i has the additional benefit of providing a complete blood count with a three-part white blood cell differential and software for patient data storage and handling.

## Introduction

There are nearly 40 million people worldwide living with HIV infections, and numbers of newly infected patients reported last year were at a record high (UNAIDS, 2006). The WHO initiative ‘3by5’, launched in 2003 (WHO, 2003a), aimed to treat 3 million people with antiretroviral therapy (ART) by the end of 2005, but has unfortunately not met its targets with only around 1.33 million people receiving ART worldwide, while an estimated 6.5 million people need ART (WHO/UNAIDS, 2006). The situation remains particularly bad in poorer regions, such as sub-Saharan Africa, where ART coverage is as low as 17% (WHO/UNAIDS, 2006). The difficulty in providing adequate medical treatment is still a major problem in countries with limited resources. Very often, this is not only a matter of making financial resources and antiretroviral medications available, but also there are problems in providing access to the required medical services, not only in regional referral centres or district hospitals but also in local primary health-care centres (WHO, 2003b). While this approach has been partly successful for making antiretroviral medication available in certain countries of sub-Saharan Africa, laboratory tests used for the staging and monitoring of HIV patients are still not commonly available in local health-care centres (Baggaley, Garnett & Ferguson, 2006; The Global Fund, 2007; WHO/UNAIDS, 2007). The current method used for staging HIV infection in settings with limited resources is the sole measurement of CD4^+^ T cells (‘CD4 test’). WHO recommends a cut-off value of 200–350 CD4^+^ T cells/*μ*l for adults; patients with values below this should receive ART (WHO, 2003b). In children, the absolute CD4^+^ T-cell numbers can vary considerably and is therefore not a suitable staging marker. It is recommended by WHO and the Centers for Disease Control that the percentage of CD4^+^ T cells should be used for children under 5 years of age. The suggested cut-off values are 25% for infants up to 11 months of age, <20% for children up to 3 years and <15% for children between 3 and 5 years of age (Centers for Disease Control, 1994; HIV Paediatric Prognostic Markers Collaborative Study, 2005; WHO, 2006).

The CD4 count is usually routinely performed using flow cytometry, but these instruments are generally only available in regional referral centres because of their expense and the skill needed to operate them. Frequently, blood samples have to be transported over long distances, so samples received are not in an ideal condition for CD4^+^ T-cell measurements because of their age and there is often considerable delay of up to 3 weeks in the results being available to the referring centre. There is an urgent need for a CD4 test that can be performed in a primary health-care centre, using inexpensive, simple-to-use, point-of-care equipment. In addition, as the management and transmission of patient and laboratory data are still a problem in resource-limited settings, it would be desirable to have complementary software that allows simple data management and transmission of the laboratory report via e-mail.

The T4 Quant kit from Invitrogen (Carlsbad, CA, USA, formerly Dynal Biotech) has been a manual alternative for CD4 testing for some years (Diagbouga *et al.*, 2003; Bi *et al.*, 2005). The CD4^+^ T-cell concentration can be quantitated by a simple and inexpensive procedure, using magnetic beads coated with appropriate antibodies to separate CD4^+^ T cells from whole blood samples. A major advantage of this procedure is that it requires no complex technical equipment but on the downside it does not quantitate other cell populations or offer any flexibility in the data output. As it cannot supply a complementary leucocyte count there is no possibility of evaluating the number of CD4^+^ T cells as a percentage, which is highly recommended for children by the WHO.

A potential solution to this is to perform a CD4 count on a simple inexpensive point-of-care haematology analyser. With this, the laboratory has the opportunity to run both the three-part differential blood count and the CD4 count on one instrument without the need to purchase additional flow cytometric equipment, which should contribute to reduced costs. As it is not possible to count the CD4^+^ T cells directly on the analyser while they are still attached to the magnetic beads, the method has been modified. Firstly, it is crucial to remove the CD4^+^ monocytes from the sample, so that they do not interfere with the CD4^+^ lymphocyte count. This is carried out by binding them to CD14 covered magnetic beads and removing them on the magnetic rack. After that, all remaining CD4^+^ cells in the sample (lymphocytes only) will get isolated by the use of CD4^+^ magnetic beads. They are then incubated with a specific lysis solution (supplied) so that the CD4^+^ T cells lyse and release their nuclei. The beads are collected with a magnet and discarded. The remaining fluid containing the nuclei of the CD4^+^ T cells can then be counted on the Sysmex pocH-100i haematological analyser. The pocH-100i is a compact automated haematology analyser designed specifically for point-of-care testing (Briggs *et al.*, 2003). As well as counting the CD4^+^ nuclei, the analyser provides a full blood count and a three-part differential leucocyte count, the results of which are valuable for monitoring the possible side effects of ART.

We have developed and evaluated the performance of the T4 Quant kit in a modified format optimised for automated cell counting on the pocH-100i, using dedicated software. Linearity, stability and lot-to-lot reagent variation have been evaluated for the new system and the CD4^+^ T-cell counts were compared with a flow cytometric method as the designated comparison method. This study demonstrates a simple and robust procedure for the isolation and counting of CD4^+^ T cells.

## Materials and methods

One hundred and fifteen residual EDTA blood samples from HIV-positive patients and 21 samples from patients with leukaemia or lymphoma were obtained from Haematology Department, University College London Hospital, UK. Measurements were performed on the day of the phlebotomy or the next day.

### Isolation and counting of the CD4^+^ T cells

**Figure 1:**
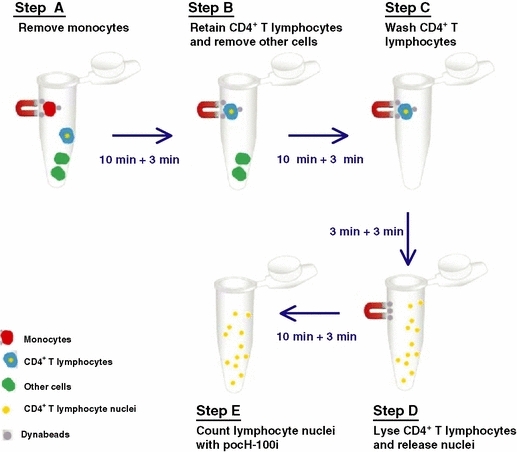
Flow chart of the immunomagnetic isolation procedure. After the removal of monocytes with anti-CD14 covered magnetic beads (step A), anti-CD4^+^ covered immunomagnetic beads are used to isolate CD4^+^ T cells from whole blood samples (step B). The CD4^+^ T cells are washed and lysed in the same tube and finally the free nuclei are counted on the pocH-100i haematology analyser to estimate the number of CD4^+^ T cells/*μ*l blood.

Isolation and counting of the CD4^+^ T cells require the Dynal T4 Quant Kit/Reagent Kit for Sysmex Analysers (Invitrogen, Oslo, Norway), and the components of the Sysmex CD4 system, namely: a pocH-100i with k-xpert CD4 data management software for appropriate calculation of CD4^+^ T cells (Sysmex Europe, Norderstedt, Germany), a tube rotator, a microcentrifuge, a magnet rack, two pipettes (50 and 250 *μ*l) and capped plastic tubes (1.5 ml). The isolation procedure was carried out according to the manufacturer’s protocol and as demonstrated in Figure 1. In brief, 250 *μ*l EDTA blood was diluted with 250 *μ*l washing buffer and incubated with 50 *μ*l of magnetic beads coated with an anti-CD14 antibody (CD14 Dynabeads®) for 10 min. The beads with the monocytes attached were then removed by incubation on the magnet rack for 3 min (step A). A total of 500 *μ*l of the monocyte-free supernatant was incubated with 50 *μ*l CD4 Dynabeads® on the tube rotator for 10 min at 50 rpm (step B), then the beads with the CD4^+^ T cells attached were separated and washed on the magnet rack (2 × 3 min, step C). The tube was removed from the rack and 100 *μ*l lysis solution was added to release the nuclei of the CD4^+^ T cells (10 min). After lysis, the tube was again placed in the magnet rack to remove the beads (3 min, step D). The supernatant was transferred into a fresh tube and the nuclei of the CD4^+^ T cells were counted on the pocH-100i in the white blood cell (WBC) channel (step E).

**Figure 2:**
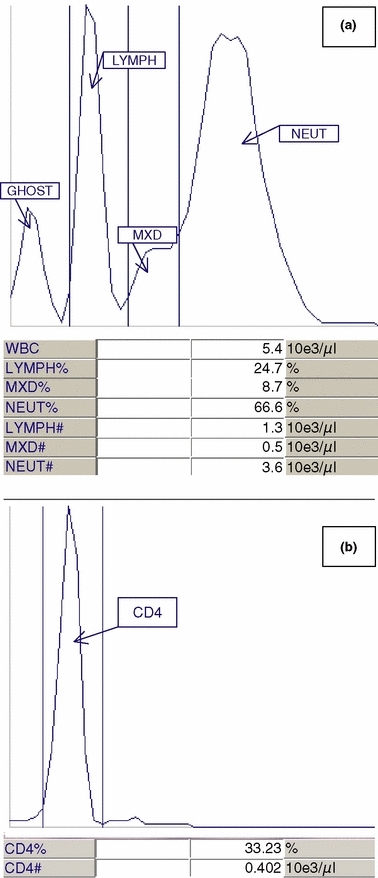
Screenshots depicting the histograms and numerical results for WBC (a) and CD4^+^ T-cell (b) count from one blood sample. MXD = the sum of monocytes, eosinophils and basophils.

By performing a complete blood count (CBC) on the same instrument, it is possible to measure both, the absolute CD4^+^ T-cell concentration and indirectly the corresponding value as a percentage of the total lymphocyte concentration. Representative histograms and data from one sample are shown in Figure 2. By using the full capacity of the magnetic racks, up to 12 patient samples can be analysed per hour by a single experienced operator.

### Imprecision

Blood samples with varying concentrations of CD4^+^ T cells were used to assess imprecision. To asses the within-run imprecision of the final measurement step alone, five blood samples with WBC concentrations between 1600 and 100 cells/*μ*l were measured 10 times consecutively on the pocH-100i haematology analyser. The total imprecision of the method was then measured by selecting five different blood samples with varying CD4^+^ T-cell concentrations (between 70 and 855 cells/*μ*l), estimated by flow cytometry. Each of these samples were split into 10 aliquots and the CD4^+^ T-cell nuclei were isolated according to the immunomagnetic separation protocol and counted in triplicate on the pocH-100i.

### Linearity

Linearity was measured according to the ICSH (1994) guidelines. A blood sample containing 1660 CD4^+^ T cells/*μ*l was serially diluted up to 16-fold. The CD4^+^ T-cell nuclei were measured in triplicate for each dilution.

### Sample stability

Three blood samples were selected with varying concentrations of CD4^+^ T cells. Each sample was aliquoted into three tubes which were then stored at 4, 20 and 37 °C, respectively. The isolation and measurement was performed immediately and after 24 and 48 h.

### Methods comparison

The methods comparison comprised 115 samples from HIV-positive patients. CD4^+^ T-cell concentrations as quantitated by flow cytometry ranged from 7 to 1392/*μ*l. All samples were analysed by immunomagnetic isolation of CD4^+^ T cells with subsequent measurement on the pocH-100i and compared with two different flow cytometric methods: Beckman Coulter Epics® XL-MCL™ Flow Cytometer, system ii software, version 3 using the Cyto-stat tetraCHROME CD45-FITC/CD4-RDI/CD8-ECD/CD3-PC5 reagent kit and the tetraone system software version 1 (all Beckman Coulter, Miami, FL, USA) and BD FACSCalibur using cellquest software and BD CD45/CD3/CD4/CD8 monoclonal antibodies (Beckton Dickinson, Franklin Lakes, NJ, USA). The data were compared by statistical correlation analysis using Spearman’s (1904) rank correlation coefficient. Regression equations were calculated according to Passing & Bablok (1983); agreement was assessed with Bland–Altman plots.

### Lot-to-lot reagent variation

Fifty-four patient samples were measured simultaneously using two different lots of the Dynal T4 Quant kit/Reagent kit for Sysmex analysers.

### Effect of abnormal lymphocytes on the assay

To assess whether abnormal lymphocytes affect the manual isolation step 21 samples from patients with leukaemia or lymphoma were tested in duplicate for the immunomagnetic separation of CD4^+^ T cells followed by measurement on the pocH-100i and compared with counts from the Epics XL flow cytometric method.

## Results

### Imprecision

The results show acceptable within-run imprecision with a coefficient of variation (CV) between 3.9% and 8.1% for concentrations of 100–1600 CD4^+^ T cells/*μ*l. The total imprecision of the method, isolation of the nuclei and counting on the pocH-100i demonstrated increasing imprecision with lower CD4^+^ T-cell numbers; the CV was, however, still only about 16% at the cut-off level of 200 CD4^+^ T cells/*μ*l and about 12% at 350 CD4^+^ T cells/*μ*l, as demonstrated in Table 1.

**Table 1:** Concentration dependence of the CV of immunomagnetic isolation with subsequent measurement of CD4^+^ T-cell nuclei on the Sysmex pocH-100i

CD4^+^ T cells/*μ*l whole blood	CV (%)
27.7	18.0
135.8	13.8
198.1	16.3
384.6	11.3
654.4	11.6

CV, coefficient of variation. The determined imprecision includes the imprecision of the manual isolation step plus that of the measurement.

### Linearity

**Figure 3:**
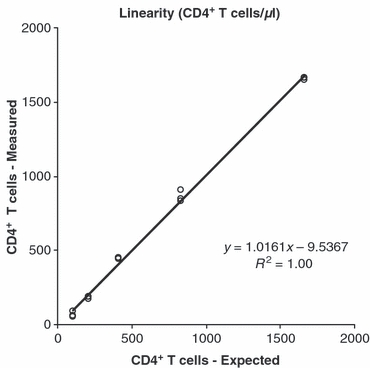
Linearity of the immunomagnetic isolation and subsequent measurement of the CD4^+^ T-cell nuclei on the pocH-100i (coefficients were calculated by linear regression).

The manual isolation and counting of CD4^+^ T-cell nuclei demonstrated excellent linearity in the evaluated range between 100 and 1600 cells/*μ*l (Figure 3).

### Stability

**Figure 4:**
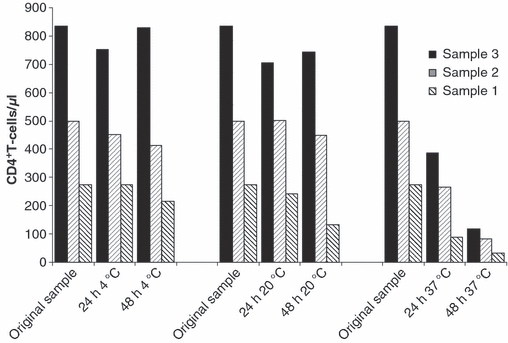
Demonstration of sample stability of samples after storage for up to 48 h at 4, 20 and 37 °C, respectively.

Results for stability are demonstrated in Figure 4. Samples were stable for 24 h when stored at 4 or 20 °C with an unsteady decline of CD4^+^ T-cell counts of 15% maximum, which is within the range of the expected imprecision of the method. Longer storage at these temperatures led to further decrease of CD4^+^ T-cell counts.

At increased temperature, the recovery of CD4^+^ T cells decreased over the course of time; the higher the temperature, the greater the loss of CD4^+^ T-cell nuclei.

### Methods comparison and concordance analysis

**Figure 5:**
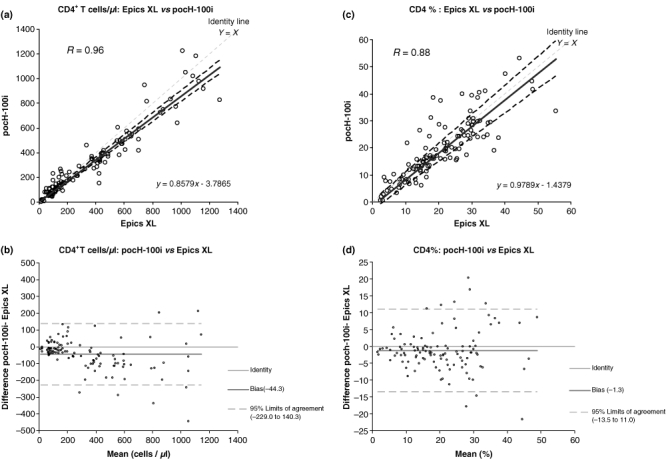
Methods comparison of immunomagnetic isolation with subsequent measurement of the CD4^+^ T-cell nuclei/*μ*l (a, b) or CD4 percentages (c, d) on the pocH-100i with flow cytometric measurements on the Epics depicted are Passing and Bablok regressions with the 95% confidence interval (dashed lines), Spearman’s rank correlation coefficient *R* and Bland–Altman plots.

Comparison of results between the immunogenic isolation with automated counting and the two flow cytometric methods demonstrates good correlation. Figure 5a,b shows the correlation analysis and Bland–Altman agreement for the comparison of the manual method and the Epics XL flow cytometer. Comparison with the FACScalibur flow cytometer produced similar results for agreement and correlation, with the Spearman’s rank correlation coefficient *R* being 0.93 for CD4^+^ T cells/*μ*l and 0.86 for CD4 percentage.

However, both flow cytometric methods demonstrated higher CD4^+^ T-cell counts than the immunomagnetic separation with pocH-100i count. On average, results from the Epics XL were 16.6% higher than the pocH-100i and 23.6% higher on the FACSCalibur. Concordance between the different methods was determined by means of contingency tables, classifying results as to whether they were higher or lower than the minimum cut-off value of 200 CD4^+^ T cells/*μ*l (Table 2a,b). Both methods picked up similar numbers of the same patients with critical CD4 values of 200/*μ*l and below (*n* = 57 and 55, respectively), or with CD4^+^ T-cell concentrations above 200/*μ*l (58 and 60, respectively). Using this cut-off value, the concordance with the Epics XL was 93.1%. With the data management software k-xpert (Droest, 2002), the pocH-100i offers the possibility for the user to introduce a calibration factor. After introducing a calibration factor of 1.166 to compensate for the generally higher values on the Epics XL, the concordance was reassessed and found to be exactly the same at 93.1%. The concordances with the results obtained with the FASCalibur were 93.4%, both with and without calibration factor (data not shown).

**Table 2:** Concordance between immunomagnetic isolation with consecutive measurement of the CD4^+^ T-cell nuclei on the pocH-100i and flow cytometric measurement on the Epics XL (a) and after application of the respective calibration factor (b)

*n* = 115	Epics XL ≤200/*μ*l	Epics XL >200/*μ*l
(a) pocH-100i *vs.* Epics XL (not calibrated) (%)		
pocH-100i > 200/*μ*l	5 (4.3)	55 (47.8)
pocH-100i ≤ 200/*μ*l	52 (45.2)	3 (2.6)
(b) pocH-100i *vs*. Epics XL (calibrated) (%)		
pocH-100i > 200/*μ*l	7 (6.1)	57 (49.6)
pocH-100i ≤ 200/*μ*l	50 (43.5)	1 (0.9)

Concordance 93.1%. Discordance 6.9%.

As it is more advisable to estimate the percentage of CD4^+^ T cells (CD4 percentage) in patients <5 years of age, rather than the absolute count, we tested whether the pocH-100i method could allow for this paradigm. We assessed the CD4 percentage from the immunomagnetic isolation with automated counting and the internal total lymphocyte count from the pocH-100i and compared it with the CD4 percentage data from the Epics XL and the FACSCalibur. Figure 5c,d demonstrates the correlation and agreement for the manual method and the Epics XL.

### Lot-to-lot reagent variation

**Figure 6:**
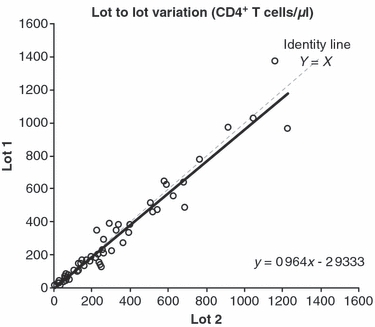
Lot-to-lot reagent variation of immunomagnetic isolation with measurement of the CD4^+^ T-cell nuclei on the pocH-100i.

Two different lots of Dynal magnetic beads and reagents were used to determine CD4^+^ T-cell concentrations. Both of them demonstrated comparable results on all samples (Figure 6).

### Leukaemia and lymphoma samples

We also quantified CD4^+^ cells on 21 blood samples from patients with leukaemia or lymphoma by immunomagnetic isolation and counting on the pocH-100i and compared the results with those generated on the Epics XL flow cytometer (Table 3). Although, with an average CV value of 4%, the reproducibility of the immunomagnetic separation was as good as with samples from HIV patients (data not shown), results were often quite diverse. This was particularly true for patients with a high lymphocyte concentration and highly abnormal cells present.

**Table 3:** Comparison of immunomagnetic isolation with measurement of the CD4^+^ T-cell nuclei on the pocH-100i and flow cytometric measurement on the Epics XL in patients with leukaemia and lymphoma

No.	pocH-100i CD4/*μ*l blood	Epics XL CD4/*μ*l blood	Lymphocytes/*μ*l blood	Diagnosis
1	6394	47 623	50 200	T-cell lymphoma, many very atypical lymphocytes
2	950	5556	11 400	Plasma cell leukaemia, many circulating plasma cells
3	408	136	4040	Hodgkin’s disease/CLL
4	821	1855	13 190	CLL
5	785	630	7140	Hodgkin’s disease
6	1347	1385	5160	NHL
7	623	554	54 990	CLL
8	903	882	35 900	B-cell lymphoproliferative disease, not characterized
9	580	854	2610	Pre-B ALL
10	1024	1960	21 750	NHL
11	987	1279	6470	B-cell Lymphoma
12	934	1419	15 370	NHL
13	489	423	1130	Mantle cell lymphoma, many apoptotic lymphocytes,occasionally blasts
14	571	1071	2750	ALL 4% blasts
15	375	2223	8380	ALL 84% blasts
16	1114	Not gateable	22 650	AML 58% blasts, 22.65 × 10^9^/l lymphocytes
17	112	74	17 610	Lymphoma, many smear cells present
18	1345	1324	59 290	B-CLL 5–10% prolymphocytes
19	1054	1198	5270	AML 87% blasts
20	1737	2331	20 097	Precursor B-ALL,CD10-positive 15% blasts
21	1129	2369	8450	Precursor B-ALL, CD10-negative, 90% blasts

CLL, chronic lymphatic leukaemia; NHL, non-Hodgkin lymphoma; ALL, acute lymphatic leukaemia; AML, acute myeloid leukaemia; Not gateable, Epics XL automated software was unable to gate on the CD4^+^ population of lymphocytes. Total lymphocyte numbers were counted on the Sysmex XE-2100.

## Discussion

The measurement of CD4^+^ T cells is the most important test for the staging and therapeutic monitoring of HIV-infected patients in resource-limited settings. The lack of simple and affordable CD4 tests has been a major obstacle for bringing antiretroviral treatment to patients in more remote locations in Africa and other areas. Currently, samples are usually sent to distant regional referral centres. The quality of the blood samples is affected by the transport and time delay. In centralized testing centres, the samples are usually analysed by flow cytometry which is costly and requires skilled operators. Centralized testing results in a delay in the results being available with a consequent delay in treating the patient. In South Africa and Botswana, some of the authors observed turnaround times of 3 weeks and longer even when the testing centres are located <50 km from the primary health-care centre. Delayed reporting has a negative impact on disease management and without a means of recalling the patient for treatment when the results do arrive treatment is often not initiated. It would therefore be desirable to perform the CD4 tests at the local hospital or even at the primary health-care centres where the patient delivers a blood sample and can wait for the CD4 result and receive appropriate medical counselling and treatment if required all in one visit. As well as keeping the patient’s data on file in the primary health-care centre, it should be possible to make these data available to the remote referral centres for the purpose of surveillance, epidemiology and health-care policy.

The proposed CD4 point of care testing (POCT) system can be performed by a nurse, health worker or medical technician after the person has received appropriate training. In addition to hands-on training, the manufacturer supplies a training DVD, showing all the steps of the assay in a detailed and simple way. As the samples are processed in parallel, a trained person can perform up to 12 tests per hour. It is possible to use software developed for the system to keep track of previous haematological data of the individual patient, which is beneficial for treatment decisions by the health-care staff. For between-run imprecision, the CV has been determined to be around 16% at the WHO recommended minimum cut-off level of 200 CD4^+^ T cells/*μ*l. The test results are linear between 100 and 1660 CD4^+^ T cells/*μ*l.

The methods comparison with two flow cytometric methods demonstrated a generally good correlation over the whole concentration range. The results were slightly higher by flow cytometry as compared with immunomagnetic isolation with subsequent measurement of CD4^+^ T cell nuclei on the pocH-100i. A possible explanation is a small loss of cells during the separation procedure. Some CD4^+^ T cells might be unstable and disintegrate during the isolation procedure.

Data from the last five surveys of participants of the UK National External Quality Assurance Schemes (NEQAS) for leucocyte immunophenotyping demonstrated an inter-laboratory variability of flow cytometric CD4 tests ranging from CVs of 9% to 14% on counts from 133 to 827 CD4^+^ T cells/*μ*l.

Therefore, the deviation between the immunomagnetic isolation with subsequent measurement of CD4^+^ T-cell nuclei on the pocH-100i and the flow cytometric measurement is acceptable, especially because to date an internationally recognized reference procedure for the measurement of CD4^+^ T cells does not exist; however, a routine dual-platform flow cytometry system was selected as the designated comparison method for this study.

From a clinical point of view, concordance between the methods is important because patients are classified and scheduled for treatment or not according to whether the measurement result is above or below a protocol-defined cut-off value. The concordance between immunomagnetic isolation and consecutive measurement of CD4^+^ T-cell nuclei on the pocH-100i and flow cytometric measurement on the Epics XL and FACSCalibur was 93.1% and 93.4%, respectively. The concordance rate remained the same after the immunomagnetic isolation and measurement of CD4^+^ T-cell nuclei on the pocH-100i has been calibrated to flow cytometry. Probably because of different methodology, we found a constantly lower absolute CD4 count compared with the flow cytometers. To account for this difference, we developed a calibration factor in the k-xpert software settings. This calibration caused reclassification of four of 115 patients (3.5%) from below the cut-off value to above the cut-off value on the pocH-100i. As two of these patients were below the cut-off value on the Epics XL and two were above the overall concordance did not change. The same situation occurred for the FACSCalibur. If locally required, the immunomagnetic isolation and measurement of CD4^+^ T-cell nuclei on the pocH-100i may be calibrated to flow cytometry.

Because of the high variability of the total lymphocyte concentration in children, the WHO recommends that the absolute CD4^+^ T-cell concentration is not used for children under 5 years of age. Instead, the percentage of CD4^+^ T cells should be used (HIV Paediatric Prognostic Markers Collaborative Study, 2005; WHO, 2006). As the pocH-100i can measure the total lymphocyte concentration, it is suitable for CD4 testing in accordance with WHO recommendations for both adults and children. In addition, the method comparison studies of this work clearly show that estimations of CD4 percentages correlate well with the results from the flow cytometers.

In patients with leukaemia or lymphoma, the two methods sometimes gave quite different results. It is not possible to say which method is more accurate as the standardized gating procedure on the flow cytometer might not apply for some of these samples. In one case, automated gating was even impossible. While there is a rather low incidence of HIV patients with leukaemias, the occurrence of non-Hodgkin lymphomas (NHL) has been increasing, with HIV-positive patients being around 350 times more likely to develop an NHL (Goedert, 2000) than non-infected people. However, the pocH-100i will provide a ‘flag’ in the presence of abnormal WBC, blasts or atypical lymphocytes, which should alert operators to look at the blood film morphology of the cells and treat the CD4^+^ T-cell results with caution if lymphoma cells are present or refer the sample to a specialized regional centre.

The sample stability experiments demonstrated that the samples were stable at 4 and 20 °C for 24 h. Longer storage as well as higher temperatures led to an impaired recovery of CD4^+^ T cells. Sample stability is generally a problem for CD4 analysis, including flow cytometric measurements (Diagbouga *et al.*, 2003). For immunomagnetic isolation and measurement of CD4^+^ T-cell nuclei on the pocH-100i, the blood samples should therefore be stored in a refrigerator and measured preferably within 24 h. This should be achievable in a primary health-care centre as the whole measurement procedure is performed on site as POCT; the pre-analytical conditions can be much better controlled than in centralized testing where long distance transport can be a crucial factor. The comparison of two different reagent lots demonstrated no relevant lot-to-lot variation.

The concept of using a point-of-care haematological analyser to measure CD4^+^ T cells in primary health-care centres includes the additional benefits of having the possibility to perform a CBC and a three-part differential blood count on any patient requiring it (Figure 2). This is important because monitoring of ART requires haematological measurements as most drugs used for treatment can cause anaemia and neutropenia.

By following the ambitious WHO ‘3by5’ (WHO, 2003a; WHO/UNAIDS, 2006) initiative, the number of HIV/AIDS patients getting antiretroviral treatment is expected to increase extensively. The central organization of the data accumulating at rural health-care facilities in the future will be a great challenge which can only be handled by electronic data processing. Data from the pocH-100i can easily be transferred to regional referral hospitals or a central data bank, using the complementary data management software k-xpert, allowing for a higher standard of quality assurance. Furthermore, the k-xpert provides simple graphical and numerical tools which help to identify and combine all data for each patient which is an important point for the monitoring of ART. This method for measuring CD4^+^ lymphocytes offers an alternative to the expensive flow cytometry count with the advantage that it is rapid and simple to perform in all laboratories without the need for complex skills and equipment.
